# Potential of *Bacillus paramycoides* hydrolase to degrade propiconazole

**DOI:** 10.2478/aiht-2026-77-4001

**Published:** 2026-03-30

**Authors:** Muhammad Naveed, Maida Salah Ud Din, Tariq Aziz, Rida Naveed, Daochen Zhu, Maha Alharbi, Ashwag Shami

**Affiliations:** University of Central Punjab Faculty of Science and Technology, Department of Biotechnology, Lahore, Pakistan; Jiangsu University, Biofuels Institute; School of the Environment and Safety Engineering, Zhenjiang, China; University of Ioannina Arta, Department of Agriculture, Laboratory of Animal Health, Hygiene and Food Quality, Arta, Greece; Princess Nourah bint Abdulrahman University College of Science, Department of Biology, Riyadh, Saudi Arabia

**Keywords:** bioremediation, environmental toxicology, hydrolase enzymes, molecular docking, propiconazole degradation, bioremedijacija, enzimi hidrolaze, molekulsko dokiranje, okolišna toksikologija, razgradnja propikonazola

## Abstract

Propiconazole is a widely used synthetic fungicide that has raised deep environmental and toxicological concerns due to its persistence and bioactivity. In this study, we investigated the potential of a facultative anaerobic, Gram-positive, rod-shaped bacterium, *Bacillus paramycoides* to degrade propiconazole by elucidating the functional role of its hydrolase enzyme *in silico*. The hydrolase was characterised with the FASTA sequence to determine its physicochemical properties, stability, and conserved functional domains. Homology modelling was performed and the predicted structure validated using a Ramachandran plot and ERRAT analysis, yielding an overall quality score of 93.6 %. Eleven propiconazole-related compounds, including parent molecules and degradation products (e.g. hispor, propiconazole TP1, propiconazole TP2, and propiconazole-d7) were retrieved from the PubChem database and subjected to molecular docking using PyRx. Docking analysis revealed stable enzyme-substrate interactions, with the highest binding affinity of −6.8 kcal/mol observed for native hydrolase complexes. Site-directed mutagenesis was subsequently performed, and mutant structures were evaluated for structural stability and functional integrity. The mutated hydrolase exhibited an improved binding affinity of −7.4 kcal/mol, indicating enhanced substrate interaction. Molecular dynamics simulations using the AMBER force field further confirmed the structural stability, binding consistency, and functional reliability of the enzyme-ligand complexes. Overall, these quantitative findings support the potential of *B. paramycoides* hydrolase as a stable, non-virulent, and efficient candidate for environmentally sustainable bioremediation of propiconazole, with relevance to environmental and occupational toxicology.

## Abstract (hr)

Propikonazol je raširen sintetski fungicid koji izaziva značajnu zabrinutost zbog svoje postojanosti i bioaktivnosti, što može imati ozbiljne okolišne i toksikološke implikacije. Ovdje smo ispitivali sposobnost fakultativno anaerobne, Gram-pozitivne, štapićaste bakterije *Bacillus paramycoides* da razgradi propikonazol, točnije funkcionalnu ulogu njezina enzima hidrolaze *in silico*. Hidrolaza je okarakterizirana na temelju FASTA sekvence radi određivanja fizikalno-kemijskih svojstava, stabilnosti i očuvanih funkcionalnih domena. Provedeno je modeliranje homologije, a predviđena struktura validirana je Ramachandranovim dijagramom i ERRAT analizom, pri čemu je dobivena ukupna ocjena kvalitete modela od 93,6 %. Jedanaest spojeva povezanih s propikonazolom, uključujući matične molekule i produkte razgradnje (npr. hispor, propikonazol TP1, propikonazol TP2 i propikonazol-d7), preuzeto je iz baze podataka *PubChem* te podvrgnuto molekulskom dokiranju primjenom programa PyRx. Analiza dokiranja pokazala je stabilne interakcije između enzima i supstrata, pri čemu je najveći afinitet vezanja od −6,8 kcal/mol zabilježen za komplekse s izvornom hidrolazom. Naknadno je provedena usmjerena mutageneza, a mutirane strukture ocijenjene su s obzirom na strukturnu stabilnost i funkcionalni integritet. Mutirana hidrolaza pokazala je poboljšani afinitet vezanja od −7,4 kcal/mol, što upućuje na pojačanu interakciju sa supstratom. Simulacije molekulske dinamike putem polja sila AMBER dodatno su potvrdile strukturnu stabilnost, dosljednost vezanja i funkcionalnu pouzdanost kompleksa enzim-ligand. Kvantitativni rezultati ovog istraživanja potvrđuju mogućnosti hidrolaze bakterije *B. paramycoides* kao stabilnoga, nevirulentnoga i učinkovitoga kandidata za okolišno održivu bioremedijaciju propikonazola, s posebnim značajem za područje okolišne i profesionalne toksikologije.

Nearly three and a half million tonnes of fungicides, insecticides, and herbicides are applied worldwide each year, raising concerns about residue accumulation in agricultural products and their potential impact on human health (1). One synthetic triazole fungicide – propiconazole – developed specifically for agricultural use, has raised particular concern, as it is extensively applied to control fungal diseases in cereals, fruits, vegetables, and turf grass (2). Its chemical structure includes a triazole ring, common for systemic fungicides. While it is effective in plant protection, excessive or improper use can contaminate soil and water and affect non-target organisms, disrupt ecosystem balance, and reduce biodiversity. Residue build-up in crops may compromise food safety and promote fungicide-resistant pathogens, posing challenges for sustainable agriculture (3). Propiconazole is relatively persistent in the environment because its synthetic, complex chemical structure is not readily degraded by abiotic factors (4).

However, several bacteria have been reported to degrade propiconazole under laboratory conditions thanks to enzymes like cytochrome P450 monooxygenases and hydrolases, which are present in these bacteria (5). Hydrolase enzymes may drive propiconazole breakdown by hydrolysing susceptible (such as ether or ester) bonds and producing smaller transformation products. Specific enzymatic pathways and the resulting metabolites and their toxicities vary and require careful characterisation. Some bacteria can use propiconazole or its transformation products as carbon or energy sources, but the detailed mechanisms of degradation often differ between strains and remain underexplored (6).

Hydrolase enzymes from the *Bacillus* species, which are known for their robustness, broad substrate tolerance, and ability to hydrolyse various xenobiotic bonds without requiring complex cofactors, have been proposed as efficient biodegradation catalysts, but no study has yet investigated the potential of a hydrolase from the *Bacillus paramycoides* strains to degrade propiconazole at molecular level.

The aim of our study was to address this gap by characterising *B. paramycoides* hydrolase and evaluating its interaction with propiconazole *in silico* through docking and simulation analyses. We anticipated that this enzyme-focused approach could provide mechanistic insights that are not easily accessible from whole-cell degradation studies, particularly regarding substrate-enzyme interactions and predicted reaction behaviour under defined conditions. Moreover, because hydrolases can operate under mild environmental conditions and often do not require additional cofactors, they may be more amenable to practical bioremediation applications than oxidoreductase-dependent or whole-cell methods.

## MATERIALS AND METHODS

### Hydrolase sequence and its structure prediction

We relied on the FASTA protein sequence consisting of 317 amino acids, taken from the National Center for Biotechnology Information (NCBI, Bethesda, MD, USA) database under accession No. OJD80101.1, because hydrolases produced by *B. paramycoides* had already been associated with xenobiotic degradation, supporting its relevance for fungicide biodegradation studies (7). Hsp70 chaperone of *Escherichia coli* accession No. AAA18300.1 was conjugated with the protein to enhance the structural stability of the protein molecule.

The sequence was characterised using the ExPASy-ProtParam web tool (Swiss Institute of Bioinformatics, Lausanne, Switzerland), including the molecular weight, instability index, theoretical isoelectric point (pI), aliphatic index, and the grand average of hydropathicity (GRAVY) index, important for determining protein stability (Table 1).

**Table 1:** Physiochemical characterisation of the *Bacillus paramycoides* hydrolase enzyme

Amino acids (N)	932
Molecular weight	1869.85
Theoretical pI	5.18
Negatively charged residues (N)	127
Positively charged resides (N)	90
Formula	C_4492_H_7230_N_1258_O_1390_S_24_
Total No. of atoms	14394
Ext. coefficient	26025
Estimated half-life	30 h (mammalian reticulocytes, *in vitro*) >20 h (yeast, *in vivo*) >10 h (*Escherichia coli*, *in vivo*)
Instability index	38.07
Aliphatic index	100.71
Grand average of hydropathicity	−0.095

**Figure 1:**
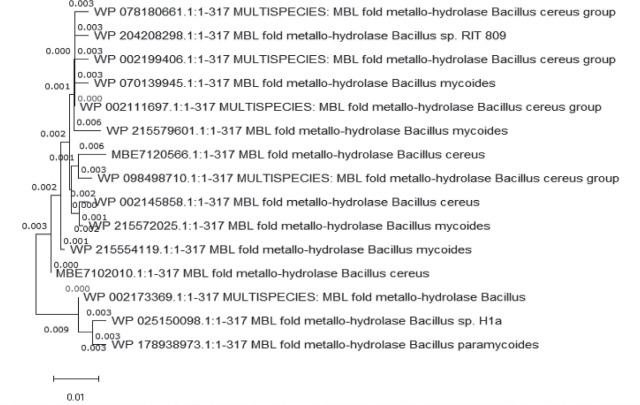
The phylogenetic tree for hydrolase

To identify regions of the sequences that remain unchanged across different samples, we ran multiple sequence alignment on the ClustalW web tool (European Bioinformatics Institute, Hinxton, UK) and analysed the results with the Molecular Evolutionary Genetics Analysis X (MEGA X) software, version 10.2.2 (Pennsylvania State University, University Park, PA, USA) to highlight the conserved portions of the sequence important for their biological function. MEGA X neighbour-joining approach and Poisson substitution method were also used to determine sequence homology and evolutionary linkages (Figure 1). This ensured that the selected hydrolase shared conserved functional motifs typical of biodegradation enzymes and justified its selection for downstream structural modelling.

To gain preliminary insight into the folding pattern of the protein prior to three-dimensional modelling, the secondary structure of the *B. paramycoides* hydrolase was predicted using the Self-Optimized Prediction Method with Alignment (SOPMA) tool, as it helps to understand the arrangement of coils, B turns, and helices. Secondary structure prediction was included to support model reliability and confirm the presence of structural elements typical for hydrolase, including α-helices, β-sheets, and loop regions that contribute to the folding pattern associated with catalytic activity in bacterial hydrolases (Table 2).

**Table 2:** Secondary structure predicted with SOPMA

**Alpha helix**	**Extended strands**	**Beta turns**	**Random coils**
41.64 %	19.24 %	7.57 %	31.55 %

**Figure 2:**
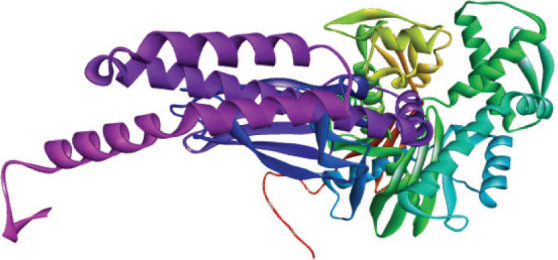
3D structure of hydrolase generated by the SWISS-MODEL tool. The purple region shows the Hsp70 chaperone of *E. coli* for the production of stable conjugate protein complex

**Figure 3:**
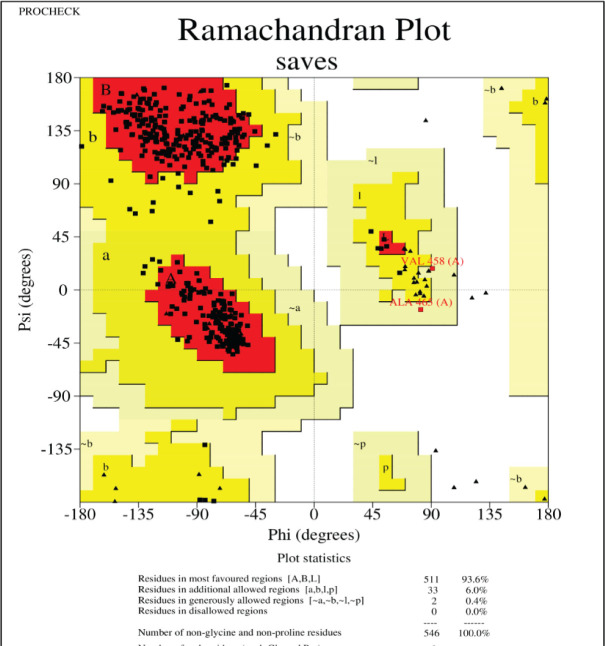
Ramachandran plot of hydrolase from *Bacillus paramycoides*

For the tertiary structure prediction we ran the primary hydrolase sequence through the SWISS-MODEL homology modelling server (Swiss Institute of Bioinformatics) (8) and selected the best out of the five predicted models for further bioinformatics analysis (Figure 2). Its structure quality was confirmed with the Ramachandran plot analysis (PROCHECK web tool, European Bioinformatics Institute) (Figure 3) (9). These steps ensured structural accuracy before docking studies.

**Figure 4:**
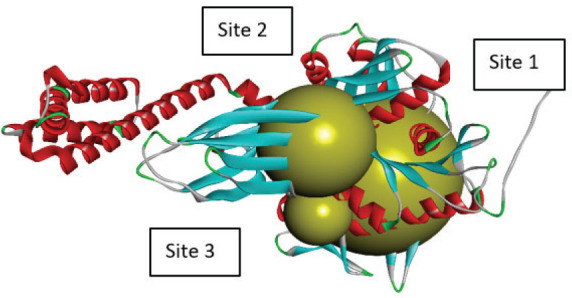
Active site prediction with Discovery Studio

The active sites of the enzyme were identified using the Discovery Studio 2025 SP1 (BIOVIA, San Diego, CA, USA) (Figure 4) and purified 3D hydrolase and ligand structures uploaded to the PyRx virtual screening tool (10), version 0.8 (Scripps Research Institute, La Jolla, CA, USA) for site-specific docking analysis. The grid box was set to the predicted dimensions (Table 3) and the result for each compound obtained in terms of free binding energy. Active-site prediction was necessary to ensure docking on biologically relevant catalytic regions rather than arbitrary pockets.

**Table 3:** Active site prediction with Discovery Studio

**Sites**	**Dimensions XYZ**	**Point Count**
Site 1	12.779000 / 8.882247 / −8.610573	5407
Site 2	−3.971000 / −3.617753 / 1.139427	1330
Site 3	−5.721000 / 12.132247 / −2.110573	708

### Docking studies

We also virtually screened structure data file (SDF) formats of 11 propiconazole compounds from the PubChem database (NCBI) (11) (Table 4) on PyRx to ensure that interaction with the hydrolase is not impeded (energy minimisation). Then 11 propiconazole models were converted into autodock ligand (pdbqt) files to identify the most stable propiconazole conformers for reliable interaction assessment with the hydrolase.

**Table 4:** Selected propiconazole and its derivatives

**Ser. no.**	**Compound name**	**PubChem CID**	**Formula**	**Structure**
1	(2R,4S)-2-(2,4-Dichlorophenyl)- 4-propyl-2-[(1H-1,2,4-triazol-1-yl) methyl]-1,3-dioxolane	679162	C_15_H_17_Cl_2_N_3_O_2_	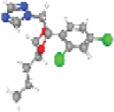
2	1-[[(2S,4S)-2-(2,4-dichlorophenyl)- 4-propyl-1,3-dioxolan-2-yl]methyl]- 1,2,4-triazole	679164	C_15_H_17_Cl_2_N_3_O_2_	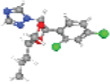
3	1-[[2-(2,4-Dichlorophenyl)-4- (2,2,3,3,3-pentadeuteriopropyl)-1,3- dioxolan-2-yl]methyl]-1,2,4-triazole	129318213	C_15_H_17_Cl_2_N_3_O_2_	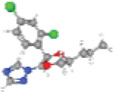
4	Hispor	156985	C_24_H_26_Cl_2_N_6_O_4_	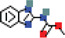 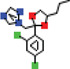
5	Propiconazole 4,4′-dihydroxybiphenyl	86643422	C_27_H_27_Cl_2_N_3_O_4_	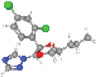
6	Propiconazole hydrochloride	129773016	C_15_H_18_Cl_3_N_3_O_2_	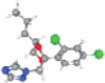
7	Propiconazole TP1	155884399	C_13_H_11_Cl_2_N_3_O_4_	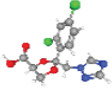
8	Propiconazole TP2	703104	C_10_H_9_Cl_2_N_3_O	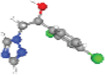
9	Propiconazole-(phenyl-d3)	124202653	C_15_H_17_Cl_2_N_3_O_2_	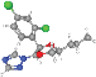
10	Propiconazole	43234	C_15_H_17_Cl_2_N_3_O_2_	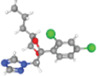
11	Propiconazole-d7	71751781	C_15_H_17_Cl_2_N_3_O_2_	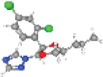

After docking with PyRx, interactions were predicted with the PyMOL Molecular Graphics System, version 2.6.2 (Schrödinger, New York, NY, USA) to identify the most favourably docked complex (12). This analysis provided insight into specific amino acid residues that form hydrogen bonds and have hydrophobic interactions with propiconazole supporting the proposed enzymatic degradation mechanism.

### Site-directed mutagenesis analysis

To determine how natural mutations would affect the hydrolase degradation effects on propiconazole we ran the *B. paramycoides* FASTA sequence through the NCBI BLASTp query and identified the closest 10 sequences of other bacterial species with a 97–100 % match. These were pairwise-aligned, and their mutations recorded on specific positions manually. Then we analysed the effect of each mutation on the original protein structure using the MuPro, Phd-SNP, I-Mutant, and SIFT tools as described elsewhere (13). By predicting which mutations would have neutral, increased, or deleterious effect, we hoped to get an insight into the enzyme’s functional resilience and identify which variants could enhance or compromise propiconazole degradation in environmental settings.

Then we generated the 3D structure of a mutated protein complex with the SWISS-MODEL and docked it to the best three of 11 propiconazole derivatives using the Autodock Vina (Scripps) for molecular docking and Discovery Studio (BIOVIA) to study their interactions and bonds and assess how mutations would influence substrate affinity, following the procedure described in detail by Baroroh et al. (14).

### Molecular dynamics simulations

Protein-ligand molecular dynamics simulations were performed using OpenMM version 8.4.0 (SimTK, Stanford University, Stanford, CA, USA). The protein was parameterised using the AMBER ff19SB force field, while ligand parameters were generated using the General AMBER Force Field 2 (GAFF2) as described elsewhere (15). Partial atomic charges of the ligand were calculated using the AM1-BCC method implemented in the Antechamber module of AmberTools (AMBER, University of California, San Francisco, CA, USA), and the resulting molecular parameters were then used to generate topology and coordinate files with the tleap program (AMBER). The protein-ligand complex was solvated in a periodic cubic box of TIP3P water molecules with a minimum padding distance of 1.0 nm from the solute. The system was neutralised by adding Na^+^ and Cl^−^ counterions to achieve electroneutrality.

Energy was minimised over 20,000 steps to remove steric clashes, followed by equilibration under constant temperature and pressure (NPT ensemble) at 298 K and 100 kPa for 5 ns. Long-range electrostatic interactions were treated using the Particle Mesh Ewald (PME) method, and a 2 fs integration timestep was applied. Covalent bonds involving hydrogen were constrained using the SHAKE algorithm. Followed a 100 ns production simulation. Trajectory coordinates for subsequent analysis were saved every 50 ps, resulting in 2,000 frames.

Structural stability was evaluated using root mean square deviation (RMSD), residue flexibility using root mean square fluctuation (RMSF), and compactness using the radius of gyration (Rg). Principal component analysis (PCA) was performed to identify dominant collective motions of the complex.

Binding free energies were estimated using the molecular mechanics / generalised born and Poisson-Boltzman surface areas (MM/GBSA and MM/PBSA, respectively) methods to quantify interaction stability. In addition, distances between the ligand and key catalytic residues were monitored throughout the trajectory to assess dynamic maintenance of the binding pose.

## RESULTS

### Docking results and protein-ligand interactions

Table 5 shows the binding energies of *B. paramycoides* hydrolase docked to 11 propiconazole compounds, while Figures 5–12 show the interactions of docked complexes, Figure 13 the mutated structure of the hydrolase enzyme, and Figures 14–16 the interactions of the mutated hydrolase enzyme with propiconazol compounds visualised with PyMOL.

**Table 5:** Binding energies and types of intermolecular interactions of compounds with hydrolase

**Compounds**	**Molecular weight (g/mol)**	**Energy (kcal/mol)**
1-[[(2S,4S)-2-(2,4-dichlorophenyl)-4-propyl-1,3-dioxolan-2-yl] methyl]-1,2,4-triazole	342.2	−6.8
Propiconazole TP1	344.15	−6.6
1-[[2-(2,4-Dichlorophenyl)-4-(2,2,3,3,3-pentadeuteriopropyl)-1,3-dioxolan-2-yl]methyl]-1,2,4-triazole	347.2	−6.2
Propiconazole	342.2	−6.2
(2R,4S)-2-(2,4-Dichlorophenyl)-4-propyl-2-[(1H-1,2,4-triazol-1-yl)methyl]-1,3-dioxolane	342.2	−6.1
Hispor	533.4	−6.1
Propiconazole-d7	349.3	−6.0
Propiconazole 4,4′-dihydroxybiphenyl	528.4	−5.9
Propiconazole hydrochloride	378.7	−5.9
Propiconazole-(phenyl-d3)	345.2	−5.8
Propiconazole TP2	258.1	−5.7

**Figure 5:**
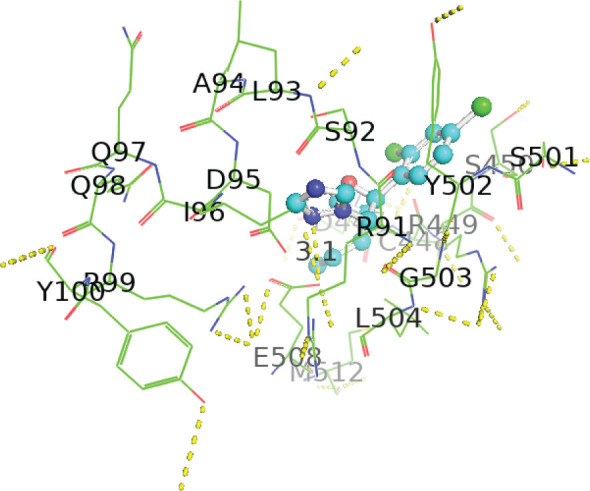
Interaction between hydrolase and (2R,4S)-2-(2,4-dichlorophenyl)-4-propyl-2-[(1H-1,2,4-triazol-1-yl)methyl]-1,3-dioxolane complex 1 visualised with PyMOL

**Figure 6:**
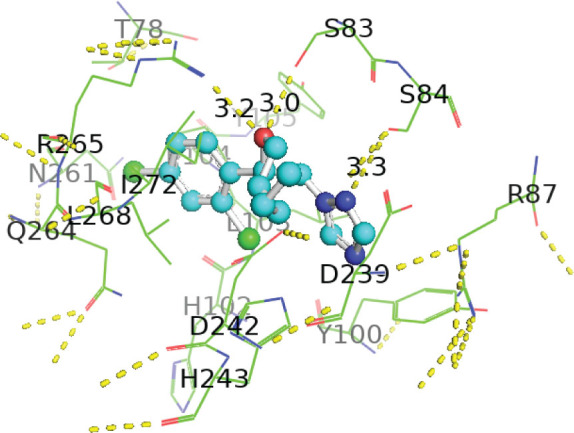
Interaction between hydrolase and 1-[[2-(2,4-dichlorophenyl)-4-(2,2,3,3,3-pentadeuteriopropyl)-1,3-dioxolan-2-yl]methyl]-1,2,4-triazole complex 2 visualised with PyMOL

**Figure 7:**
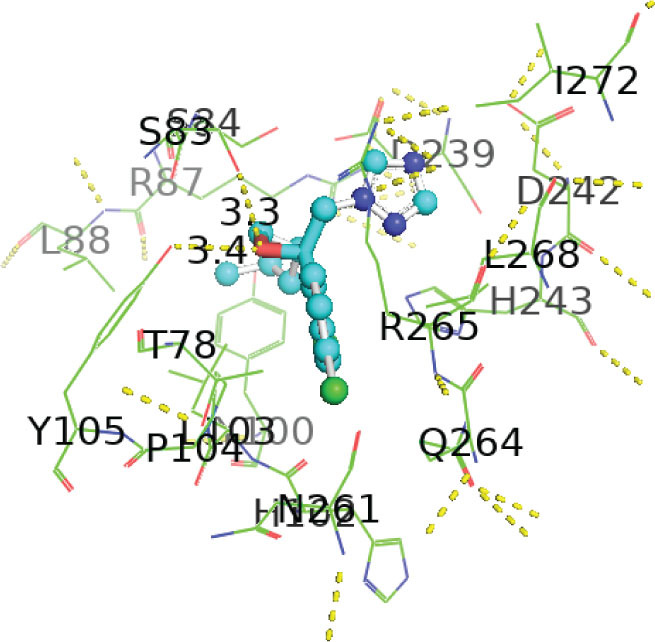
Interaction between hydrolase and 1-[[(2S,4S)-2-(2,4-dichlorophenyl)-4-propyl-1,3-dioxolan-2-yl]methyl]-1,2,4-triazole complex 3 visualised with PyMOL

**Figure 8:**
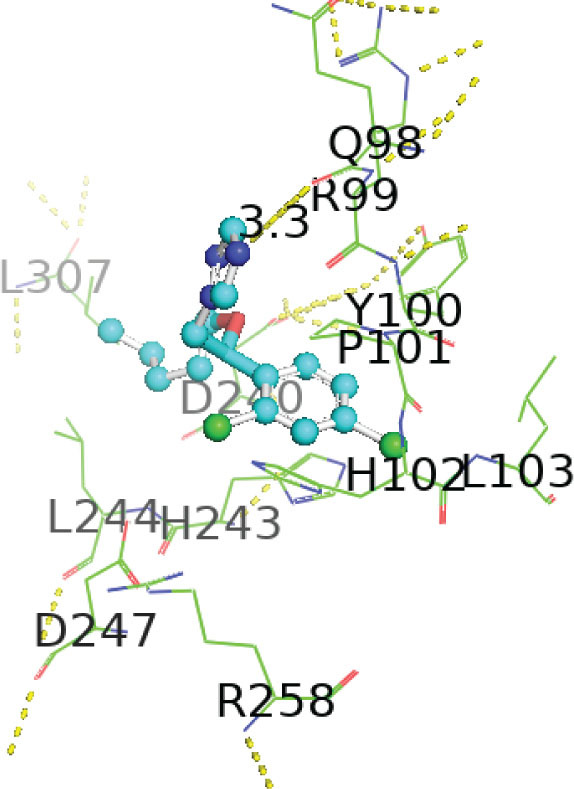
Interaction between hydrolase and propiconazole 4,4’-dihydroxybiphenyl complex 4 visualised with PyMOL

**Figure 9:**
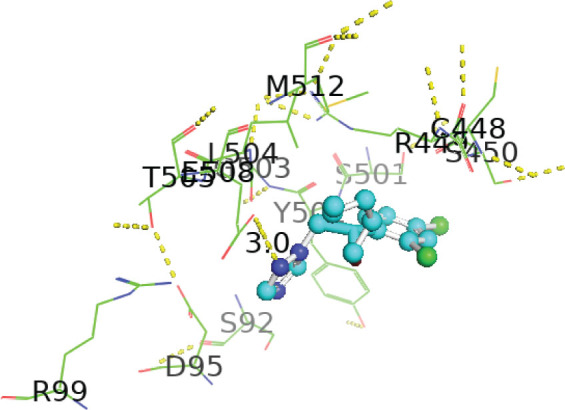
Interaction between hydrolase and propiconazole hydrochloride complex 5 visualised with PyMOL

**Figure 10:**
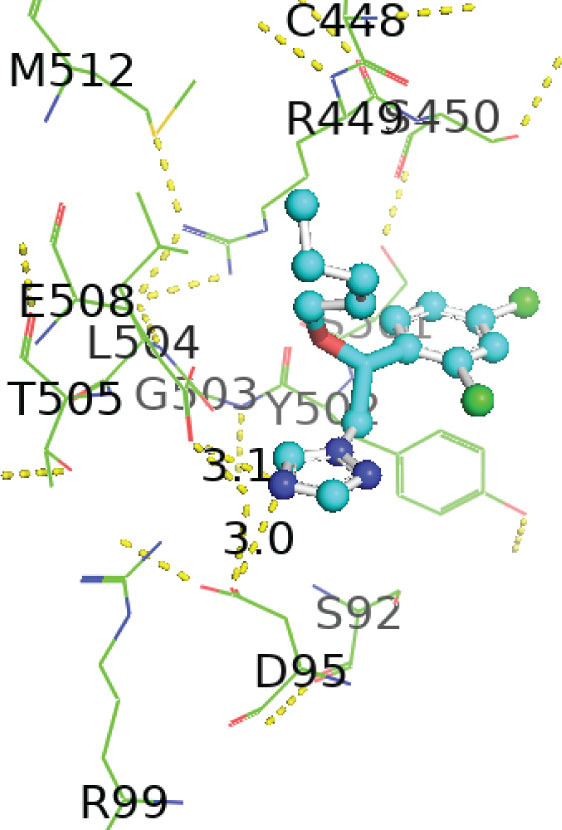
Interaction between hydrolase and propiconazole-d7 complex 6 visualised with PyMOL

**Figure 11:**
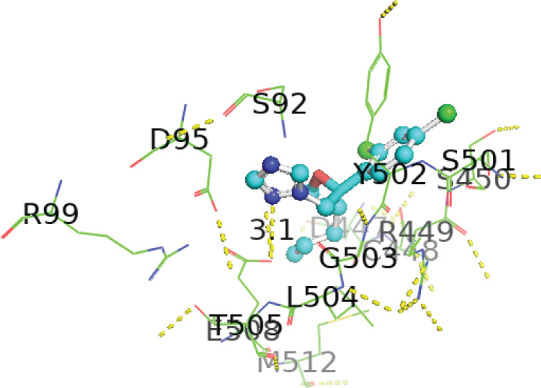
Interaction between hydrolase and propiconazole complex 7 visualised with PyMOL

**Figure 12:**
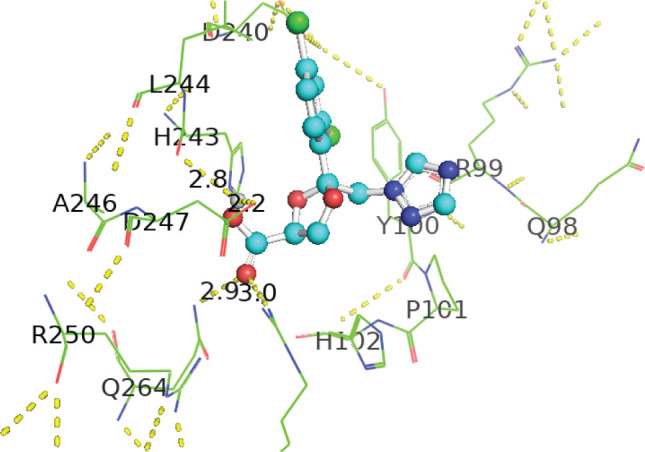
Interaction between hydrolase and propiconazole TP1 complex 8 visualised with PyMOL

**Figure 13:**
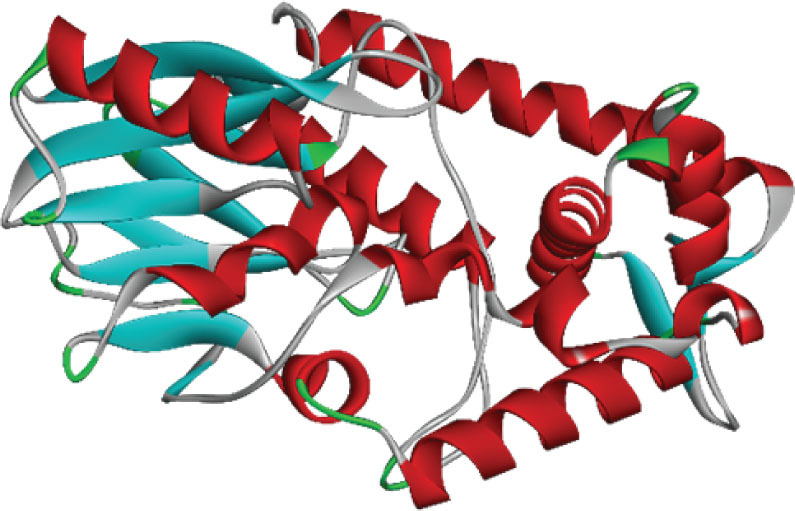
Mutated structure of hydrolase from *Bacillus paramycoides*

**Figure 14:**
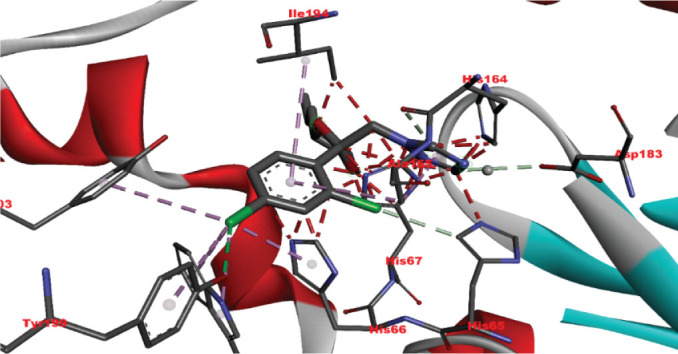
Interaction between hydrolase and propiconazole TP1. The ligand is positioned within the active site and stabilised by key catalytic residues, including His66, His67, and Trp92. Additional residues such as Phe13, Val15, Lys130, and Thr131 contribute through hydrophobic contacts and hydrogen bonding. Green lines represent hydrogen bonds, magenta lines hydrophobic and π–π interactions

**Figure 15:**
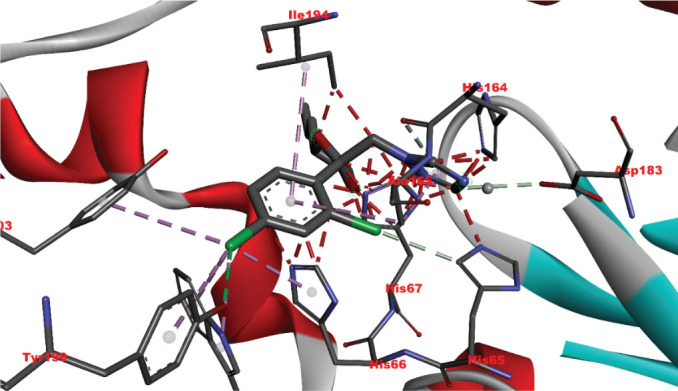
Interaction between mutated hydrolase and 1-[[2-(2,4-dichlorophenyl)-4-(2,2,3,3,3-pentadeuteriopropyl)-1,3-dioxolan-2-yl]methyl]-1,2,4-triazole

**Figure 16:**
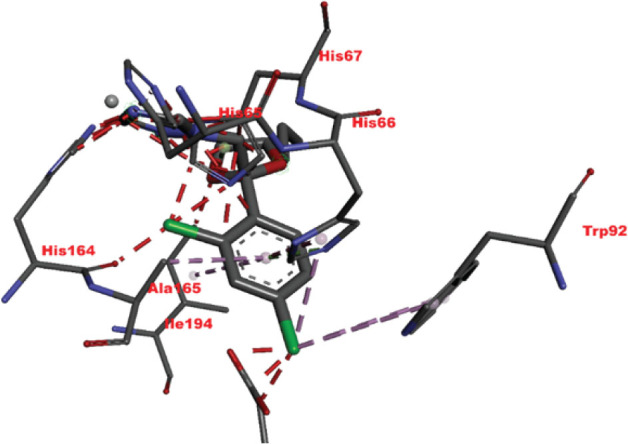
Interaction between mutated hydrolase and 1-[[(2S,4S)-2-(2,4-dichlorophenyl)-4-propyl-1,3-dioxolan-2-yl] methyl]-1,2,4-triazole

Table 6 shows how each mutation would influence the degradation effect of *B. paramycoides* hydrolase on propiconazole, and Table 7 the best docking energies (binding affinities, ranging from −7.1 to −7.4 kcal/mol) with three propiconazole variants obtained with the modelled mutated 3D structure predicted to increase the effect of the original structure. The mutated complex of hydrolase is more stable than the original structure, which suggests that mutation can enhance hydrolase functionality, stability, and interaction and can be utilised to improve degradation of propiconazole.

**Table 6:** Identified mutations and classification of mutants *in silico*

**Metallo-ß-lactamase fold metallo-hydrolase by species**	**Accession number**	**Amino acid substituted**	**Mutation position**	**Amino acid replaced with**	**I-mutant results**	**MU-PRO result**	**PHD-SNP results**	**SIFT results**
*Bacillus paramycoides*	WP_178938973.1	D	151	E	Increase	Increase	Neutral	Neutral
*Bacillus cereus*	WP_193645052.1	N	82	K	Decrease	Decrease	Deleterious	Deleterious
*Bacillus mycoides*	WP_215554119.1	V	238	M	Decrease	Decrease	Deleterious	Neutral
*Bacillus thuringiensis*	WP_264539129.1	D	123	G	Decrease	Decrease	Neutral	Deleterious
*Bacillus nitratireducens*	WP_044737773.1	L	312	W	Decrease	Increase	Neutral	Deleterious
*Bacillus* sp. CDB3	WP_128853480.1	H	47	Q	Decrease	Increase	Deleterious	Neutral
*Bacillus* sp. TH12	WP_201056898.1	A	119	V	Increase	Increase	Neutral	Neutral
*Bacillus* sp. NP247	WP_219920057.1	V	50	A	Decrease	Decrease	Deleterious	Deleterious
*Bacillus* sp. MYb209	WP_105584583.1	T	33	I	Decrease	Decrease	Neutral	Neutral
*Bacillus toyonensis*	WP_097999873.1	K	108	R	Decrease	Decrease	Neutral	Neutral

**Table 7:** Docking and interaction study of hydrolase with the best three propiconazole derivatives

**Pollutants**	**Docking energies (kcal/mol)**	**Amino acid residues**	**Distance between interacting amino acid residues (Å)**	**Type of bond interaction**
Propiconazole TP1	−7.4	HIS66, TRP92, HIS67, VAL15, THR131, PHE13, LYS130	2.84, 2.93, 3.14, 4.26, 5.01, 4.92, 4.67, 4.99, 5.23, 3.02	Alkyl, pi-alkyl, conventional, Pi-Pi T-shaped, hydrogen bonds, Van der Waals forces
1-[[(2S,4S)-2-(2,4-dichlorophenyl)-4-propyl-1,3-dioxolan-2-yl] methyl]-1,2,4-triazole	−7.1	HIS67, HIS66, TRP92, ILE192, ALA165, HIS164, HIS65	3.94, 4.85, 4.71, 4.31, 4.57, 3.68	Pi-lone pair, Pi-alkyl, Pi-Pi T-shaped, Van der Waals forces
1-[[2-(2,4-Dichlorophenyl)-4-(2,2,3,3,3-pentadeuteriopropyl)-1,3-dioxolan-2-yl]methyl]-1,2,4-triazole	−7.1	ILE194, HIS164, ASP183, ALA165, TYR139 HIS67, HIS66, HIS65,	4.42, 2.95, 2.75, 4.44, 5.50, 5.41, 3.76, 4.82, 2.70, 3.61, 2.08	Alkyl, pi-alkyl, conventional, Pi-Pi T-shaped, carbon, Waals forces hydrogen bonds, Van der

### Molecular dynamic simulation findings

**Figure 17:**
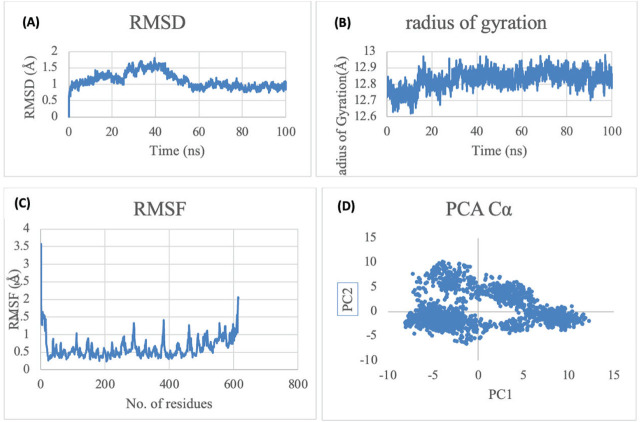
Protein–ligand complex analysis in molecular dynamics simulation. A) Root mean square deviation (RMSD) shows the overall structural stability of the complex by tracking backbone fluctuations over time. B) Radius of gyration (Rg) shows the compactness and structural integrity of the protein; consistent Rg values suggest a stable and well-folded conformation. C) Root mean square fluctuation (RMSF) highlights residue-specific flexibility, identifying regions with higher mobility such as loops, binding pockets, or terminal ends. D) Principal component analysis (PCA) illustrates dominant collective motions and major conformational transitions of the complex, helping to visualise large-scale structural dynamics that influence ligand binding and enzyme function

Figure 17 shows that the complex is stable (RMSD; panel A) and compact (radius of gyration; panel B) with little fluctuation towards 2.0 Å at 600 ns (RMSF; panel C). The RMSF profile, plotted against residue number, indicates that fluctuations are primarily confined to flexible loop regions, while residues forming the binding pocket exhibit comparatively low mobility. The fact that the two principal components (PC1 and PC2) remain in the range of −5–10 throughout the simulation suggests that the system will maintain relatively stable global motions while undergoing smaller, localised movements detailed in Figure 17 (panel D).

**Figure 18:**
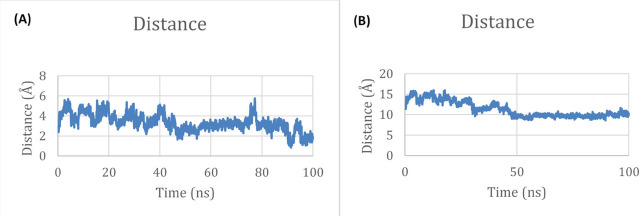
Distance analysis between the ligand and key protein residues in molecular dynamics simulation. A) Ligand-catalytic residue distances are consistently short (0.98–2.89 Å), which indicates stable interactions, essential for catalytic activity and effective binding. B) Significantly larger separation between the ligand and selected, non-catalytic residues (25.83–28.99 Å) confirms that these residues do not directly participate in ligand interaction and remain structurally distant throughout the simulation

Distance monitoring between the ligand and catalytic residues throughout the simulation confirms that the ligand remains stable in the predicted active site (Figure 18). The sustained short-range proximity indicates persistent functional interactions, supporting the structural validity of the docking pose under dynamic conditions. In contrast, residues 78, 84, and 85 maintain substantially larger distances from the ligand, confirming that they are not involved in substrate stabilisation. This comparative analysis reinforces the correct identification of the catalytic pocket and demonstrates that propiconazole remains confined within the functional binding region during the simulation.

The binding free energy calculations using MM/GBSA and MM/PBSA (Tables 8 and 9) indicate favourable protein-ligand interactions, with total binding energies of −35.91 kcal/mol and −6.75 kcal/mol, respectively. The interaction is primarily driven by van der Waals contributions, while polar solvation energy partially offsets the binding affinity.

**Table 8:** MM/GBSA complex–receptor–ligand energy decomposition

**Energy component**	**Average**	**SD**	**SEM**
VDWAALS	−39.6668	5.9813	1.8915
EEL	−0.6035	2.2259	0.7039
EGB	8.0517	2.9888	0.9451
ESURF	−3.6938	0.5431	0.1718
DELTA G gas	−40.2703	5.9055	1.8675
DELTA G solv	4.3579	3.1938	1.0100
DELTA TOTAL	−35.9124	6.4629	2.0437

MM/GBPSA – molecular mechanics / generalised born surface areas; SD – standard deviation; SEM – standard error of the mean

**Table 9:** MM/PBSA complex–receptor–ligand energy decomposition

**Energy component**	**Average**	**SD**	**SEM**
VDWAALS	−39.6668	5.9813	1.8915
EEL	−0.6035	2.2259	0.7039
EPB	12.9297	5.5557	1.7569
ENPOLAR	−21.1805	3.2240	1.0195
EDISPER	41.7699	4.0950	1.2950
DELTA G gas	−40.2703	5.9055	1.8675
DELTA G solv	33.5192	6.0584	1.9158
DELTA TOTAL	−6.7511	7.0464	2.2283

MM/PBSA – molecular mechanics / Poisson-Boltzman surface areas; SD – standard deviation; SEM – standard error of the mean

## DISCUSSION

Our findings give an important insight into the stability and reliability of the *B. paramycoides* hydrolase enzyme and demonstrate its ability to form stable complexes with the propiconazole.

The docking scores observed for the *B. paramycoides* hydrolase (−5.7 to −7.4 kcal/mol) fall well within the range of −5.0 to −8.0 kcal/mol reported for biologically active and catalytically relevant interactions, indicating that the enzyme-ligand complex exhibits a binding affinity consistent with functional degradation activity and supporting our hypothesis of the enzyme’s potential for propiconazole bioremediation (6). Our findings identify hydrogen bonding, hydrophobic interactions, and π–π stacking as major forces stabilising fungicide-enzyme complexes. Moreover, the dynamic stability observed in our AMBER molecular dynamics trajectories coincides with reported moderate structural flexibility and stable RMSD/Rg values in enzyme-ligand simulations.

Our study provides a more detailed enzyme-level mechanistic insight and additionally evaluates the effect of potential mutations through *in silico* site-directed mutagenesis described elsewhere (16), suggesting that the *B. paramycoides* hydrolase should offer a more stable and mutation-tolerant propiconazole degradation.

The addition of site-directed mutational analysis increases the research’s reliability, as incorporating likely mutations that occur over time and their impact on the stability of the enzyme-substrate complex can forecast the hydrolase’s long-term viability. Our docking simulations with mutant hydrolase (containing all mutations shown in Table 6) and propiconazole derivatives confirm that the enzyme will remain effective and robust even with mutations, bearing promise for future applications.

### Study limitations

Despite the strength of the *in silico* approaches applied, this study has several limitations. First, all structural modelling, docking, mutational analysis, and molecular dynamics simulations were computed, which means that the predicted binding affinities and the propiconazole degradation potential of *B. paramycoides* hydrolase may not fully apply in real biochemical conditions. Second, the enzymatic mechanism of propiconazole degradation was inferred from docking interactions rather than validated by wet-lab assays, so the actual catalytic pathway, rate of degradation, and potential formation of intermediate metabolites remain unconfirmed.

## CONCLUSION

Regardless of these limitations, this study provides a fair insight into the molecular and structural characteristics of *B. paramycoides* hydrolase and into its potential interaction with propiconazole. Molecular docking analysis indicates the formation of a stable enzyme-ligand complex, and site-directed mutagenesis analysis suggests that the complex should maintain this stability, despite certain amino acid substitutions. However, these predictions can only be validated by further *in vitro* and *in vivo* studies to establish the hydrolase’s practical applicability in environmental and agricultural settings. Overall, this work contributes to the broader understanding of using bacterial enzymes and formulations for sustainable bioremediation and supports research of biopesticides and biofungicides as safer alternatives to synthetic chemicals in agriculture.

## References

[1] Garraway JL, Springham DG, Moses V, Cape RE (1999). Insecticides, fungicides and herbicides. Biotechnology – The Science and the Business.

[2] Lima ÂM, Paula WTD, Leite IC, Gazolla PA, Abreu LMD, Fonseca VR, Costa AV (2022). Synthesis of eugenol-fluorinated triazole derivatives and evaluation of their fungicidal activity. J Braz Chem Soc.

[3] Goswami SK, Singh V, Chakdar H, Choudhary P (2018). Harmful effects of fungicides-current status. Int J Agric Environ Biotechnol.

[4] Chen L, Wang Z, Zhang C, Jiang W, Li X (2022). Environmental hormone effects and bioaccumulation of propiconazole and difenoconazole in *Procypris merus*. Bull Environ Contam Toxicol.

[5] Satapute P, Kaliwal B (2016). Biodegradation of the fungicide propiconazole by *Pseudomonas aeruginosa* PS-4 strain isolated from a paddy soil. Ann Microbiol.

[6] Sliti A, Singh V, Ibal JC, Jeong M, Shin JH (2024). Impact of propiconazole fungicide on soil microbiome (bacterial and fungal) diversity, functional profile, and associated dehydrogenase activity. Environ Sci Pollut Res.

[7] Ren J, Wang C, Huhetaoli, Li C, Fan B, Niu D (2020). Biodegradation of acephate by *Bacillus paramycoides* NDZ and its degradation pathway. World J Microbiol Biotechnol.

[8] Waterhouse A, Bertoni M, Bienert S, Studer G, Tauriello G, Gumienny R, Heer FT, de Beer TAP, Rempfer C, Bordoli L, Lepore R, Schwede T (2018). SWISS-MODEL: homology modelling of protein structures and complexes. Nucleic Acids Res.

[9] Carugo O, Djinović-Carugo K (2013). A proteomic Ramachandran plot (PRplot). Amino Acids.

[10] Kondapuram SK, Sarvagalla S, Coumar MS, Coumar MS Docking-based virtual screening using PyRx tool: autophagy target Vps34 as a case study. Molecular Docking for Computer-Aided Drug Design.

[11] Kim S, Thiessen PA, Bolton EE, Chen J, Fu G, Gindulyte A, Han L, He J, He S, Shoemaker BA, Wang J, Yu B, Zhang J, Bryant SH (2016). PubChem substance and compound databases. Nucleic Acids Res.

[12] Yuan S, Chan HS, Hu Z (2017). Using PyMOL as a platform for computational drug design. WIREs Comput Mol Sci.

[13] Naveed M, Shabbir MA, Aziz T, Saleem A, Naveed R, Khan AA, Alasmari AF (2023). *In silico* explorations of bacterial mercuric reductase as an ecofriendly bioremediator for noxious mercuric intoxications. Acta Biochim Pol.

[14] Baroroh U, Biotek M, Muscifa ZS, Destiarani W, Rohmatullah FG, Yusuf M (2023). Molecular interaction analysis and visualization of protein-ligand docking using Biovia Discovery Studio Visualizer. Indones J Comput Biol.

[15] Gotz AW, Williamson MJ, Xu D, Poole D, Le Grand S, Walker RC (2012). Routine microsecond molecular dynamics simulations with AMBER on GPUs. 1. Generalized born. J Chem Theory Comput.

[16] Doering JA, Lee S, Kristiansen K, Evenseth L, Barron MG, Sylte I, LaLone CA (2018). *In silico* site-directed mutagenesis informs species-specific predictions of chemical susceptibility derived from the sequence alignment to predict across species susceptibility (SeqAPASS) tool. Toxicol Sci.

